# Subtyping children with asthma by clustering analysis of mRNA expression data

**DOI:** 10.3389/fgene.2022.974936

**Published:** 2022-09-09

**Authors:** Ting Wang, Changhui He, Ming Hu, Honghua Wu, Shuteng Ou, Yuke Li, Chuping Fan

**Affiliations:** ^1^ The Affiliated Chenzhou Hospital, Hengyang Medical School, University of South China, Hengyang, Hunan, China; ^2^ Department of Pediatrics, Shenzhen Children’s Hospital, Shenzhen, Guangdong, China; ^3^ Department of Pediatrics, Chenzhou No 1 People’s Hospital, Chenzhou, Hunan, China

**Keywords:** childhood asthma, mRNA, GEO, cluster analysis, immune

## Abstract

**Background:** Asthma is a heterogeneous disease. There are several phenotypic classifications for childhood asthma.

**Methods:** Unsupervised consensus cluster analysis was used to classify 36 children with persistent asthma from the GSE65204 dataset. The differentially expressed genes (DEGs) between different asthma subtypes were identified, and weighted gene co-expression network analysis (WGCNA) was carried out. Gene Ontology and Kyoto Encyclopedia of Genes and Genomes enrichment analysis was performed for DEGs and critical gene modules. Protein–protein interactions (PPI) were constructed to obtain the hub genes. Finally, differences in the immune microenvironment were analyzed between different subtypes.

**Results:** Two subtypes (C1, C2) were identified using unsupervised consensus clustering. The DEGs between different asthma subtypes were mainly enriched in immune regulation and the release of inflammatory mediators. The important modular genes screened by WGCNA were mainly enriched in aspects of inflammatory mediator regulation. PPI analysis found 10 hub genes (DRC1, TTC25, DNALI1, DNAI1, DNAI2, PIH1D3, ARMC4, RSPH1, DNAAF3, and DNAH5), and ROC analysis demonstrated that 10 hub genes had a reliably ability to distinguish C1 from C2. And we observed differences between C1 and C2 in their immune microenvironment.

**Conclusion:** Using the gene expression profiles of children’s nasal epithelium, we identified two asthma subtypes that have different gene expression patterns, biological characteristics, and immune microenvironments. This will provide a reference point for future childhood asthma typing and personalized therapy.

## Introduction

Asthma is a heterogeneous disease (Bonnelykke and Ober, 2016; Ntontsi et al., 2021) characterized by wheezing, chest tightness, coughing, and shortness of breath (Wu et al., 2019). Because its pathogenesis is unknown, asthma is a vague term describing clinical manifestations and physiological characteristics. Several phenotypes have been proposed, including TH2-associated asthma, early-onset allergic TH2 asthma, late-onset persistent eosinophilic asthma, exercise-induced asthma, non-TH2 asthma, obesity-related asthma, and neutrophilic asthma (Wenzel, 2012). Although these phenotypes can classify asthma from certain perspectives, much controversy still remains.

The phenotype is the result of gene expression downstream, and to accurately type asthma, we still need to start at the molecular level. The term “asthma” has become obsolete, and more scholars believe that the term “asthma syndrome” should be used (Cloutier et al., 2020), while a wide range of scholars continue to explore its biological definition. Currently, with the rapid development of microarray and high throughput sequencing technologies, the research about asthma at the molecular level is rapidly developing. Yeh et al. (2018) identified three asthma subtypes based on peripheral blood mononuclear cells on microarray, the first of which had the highest eosinophil level, the second had a low eosinophil and neutrophil level, and the third showed a high neutrophil level and a poor treatment effect. Yan et al. (2015) used Affymetrix microarrays to analyze the sputum RNA sequences of asthma patients. The results showed that asthma patients could be divided into three subtypes, and different clinical manifestations and physiological characteristics were found among the three subtypes. Hekking et al. (2018) used nasal brushings, sputum, and endobronchial brushing specimens for microarray analysis of differential gene expression in children with severe asthma versus adults with severe asthma, and they found significant differences in the genetic profiles of eosinophilic airway inflammation, group 3 innate lymphocytes, lung injury, and mast cells between adults with severe asthma and children with severe asthma. These studies provide ample evidence that further typing of asthma at a molecular level holds broad potential.

Asthma is a group of diseases that are underdiagnosed and undertreated, and although the available diagnostic methods are simple and accessible, and available treatments provide symptomatic relief (Cloutier et al., 2020), the pathogenesis remains unclear. Although the incidence of asthma is increasing every year (Loftus and Wise, 2015), the causative genes are not known. Studies of asthma at the molecular level are receiving increasing attention, but few studies have been conducted to subtype asthma at the molecular level and even fewer to subtype children’s asthma at a molecular level.

In this paper we assumed that the molecular subtypes of childhood asthma can be defined by gene expression data from the nasal epithelium. Therefore, we used unsupervised consensus clustering to classify children with asthma into different subtypes and then analyzed the biological functions, immune microenvironment and hub genes of different subtypes to corroborate the nature of asthma heterogeneity. We hope to help clarify the pathogenesis of asthma and contribute to the potential targeted therapy.

## Materials and methods

### Data collection

The Gene Expression Omnibus (Barrett et al., 2013) (GEO, http://www.ncbi.nlm.nih.gov/geo/) is a public gene expression database for high-throughput microarray and next-generation sequence functional genomic data sets submitted by the research community, built and maintained by the National Center for Biotechnology Information (NCBI). The GSE65204 dataset (Yang et al., 2017), which contains nasal epithelium from 36 children with persistent asthma and 33 healthy children, was downloaded from the GEO database (Supplementary Table S1). Gene expression microarray data from 36 children with persistent asthma was selected for the next step in the analysis. RNA was sequenced using the GPL14550 chip platform. Gene probes were converted to gene symbol id, and duplicate gene probes were averaged (Supplementary Data Sheet S1). This study was based on selected data from 36 children with persistent asthma. GEO belongs to public databases. The patients whose data are in the database provided ethical approval. Users can download relevant data for free for research and publish relevant articles. Our study was based on open source data, so there were no ethical issues.

### Unsupervised consensus clustering

The ConsensusClusterPlus package (Wilkerson and Hayes, 2010) in R was used for cluster analysis, using agglomerative pam clustering with a 1-Pearson correlation distance and resampling 80% of the samples for 10 repetitions. The optimal number of clusters was determined by an empirical cumulative distribution function plot. With the first consensus clustering parameter (>0.8), we determined the number of clusters. The stats package (version 3.6.0) in R was applied to perform principal component analysis (PCA) for evaluating the differences between the different clusters.

### Differentially expressed gene screening

The limma (version 3.40.6) package in R was utilized to obtain differentially expressed genes (|Log2FC| > 1, adjusted *p*-value < 0.05) between different asthma subtypes.

### Weighted gene co-expression network analysis

The WGCNA package in R was used to construct a weighted gene co-expression network analysis. The pickSoftThreshold function was used to obtain the optimal value of the weighted parameters of adjacent functions, and the optimal value of the weighted parameters was the soft threshold for the scale-free network. The adjacency matrix was transformed into a topological overlap matrix (TOM) to estimate the distance between each gene pair. Hierarchical clustering was then performed with a dynamic approach to build clustering trees and classify genes into different modules. Finally, we assessed the correlation between asthma subtypes and each module with Pearson’s correlation analysis and identified the critical module. For further screening of hub genes in the critical module, the following conditions were set: MM is 0.8, GS is 0.2, weight is 0.15.

### Gene ontology and kyoto encyclopedia of genes and genomes pathway analysis

The clusterProfiler (version 3.14.3) package in R was used for GO and KEGG analysis, and the conditions were set that the minimum gene set was 5 and the maximum gene set was 5000, with *p*-value < 0.05. GO and KEGG analysis of DEGs and genes of the critical module selected by WGCNA were performed. The GO analysis was used to discover critical biological information about target genes, including cellular components (CC), biological processes (BP), and molecular functions (MF). KEGG is a comprehensive database that integrates genomic, chemical, and systemic functional information and is commonly used to link genomic information to high-level functional information.

### Construction for protein–protein interaction network and screening hub genes

Candidate genes were screened by overlapping between DEGs obtained by selection limma analysis, and the genes of the critical module were obtained by WGCNA. The obtained candidate genes were imported into the database (http://string-db.org), and the minimum required interaction score was set to medium confidence (0.40) with no limit on the maximum number of interactions. Cytoscape software was used for PPI visualization, and we used the MCC algorithm in the CytoHubba plugin to filter the top 10 nodes, which are considered hub genes.

### Receiver operating characteristic

We performed a ROC analysis using the R package pROC (version 1.17.0.1). At two-tailed *p*-value < 0.05 was considered statistically significant. The accuracy of the hub genes grouping in pediatric asthma patients was assessed by plotting ROC curves for hub genes.

### Immune cell infiltration analysis

The abundance of eight immune cell species and two stromal cell populations of children with asthma were calculated using the MCPCounter (Becht et al., 2016a) method, selected in the R package IOBR (Zeng et al., 2021). We compared the differences in the immunological microenvironment between the two subtypes of children. Finally, the relationships between hub genes and immune cells and stromal cells were analyzed.

### Statistical analysis

Statistical analysis was performed using R software. The t-test and means ± standard deviations were used for measures that conformed to the normal distribution. Categorical data is expressed in absolute numbers and percentages. Differences between two subtypes were tested by Student’s t-test for continuous variables, and the difference was considered statistically significant at *p*-value < 0.05.

## Results

### Unsupervised consensus clustering

**FIGURE 1:**
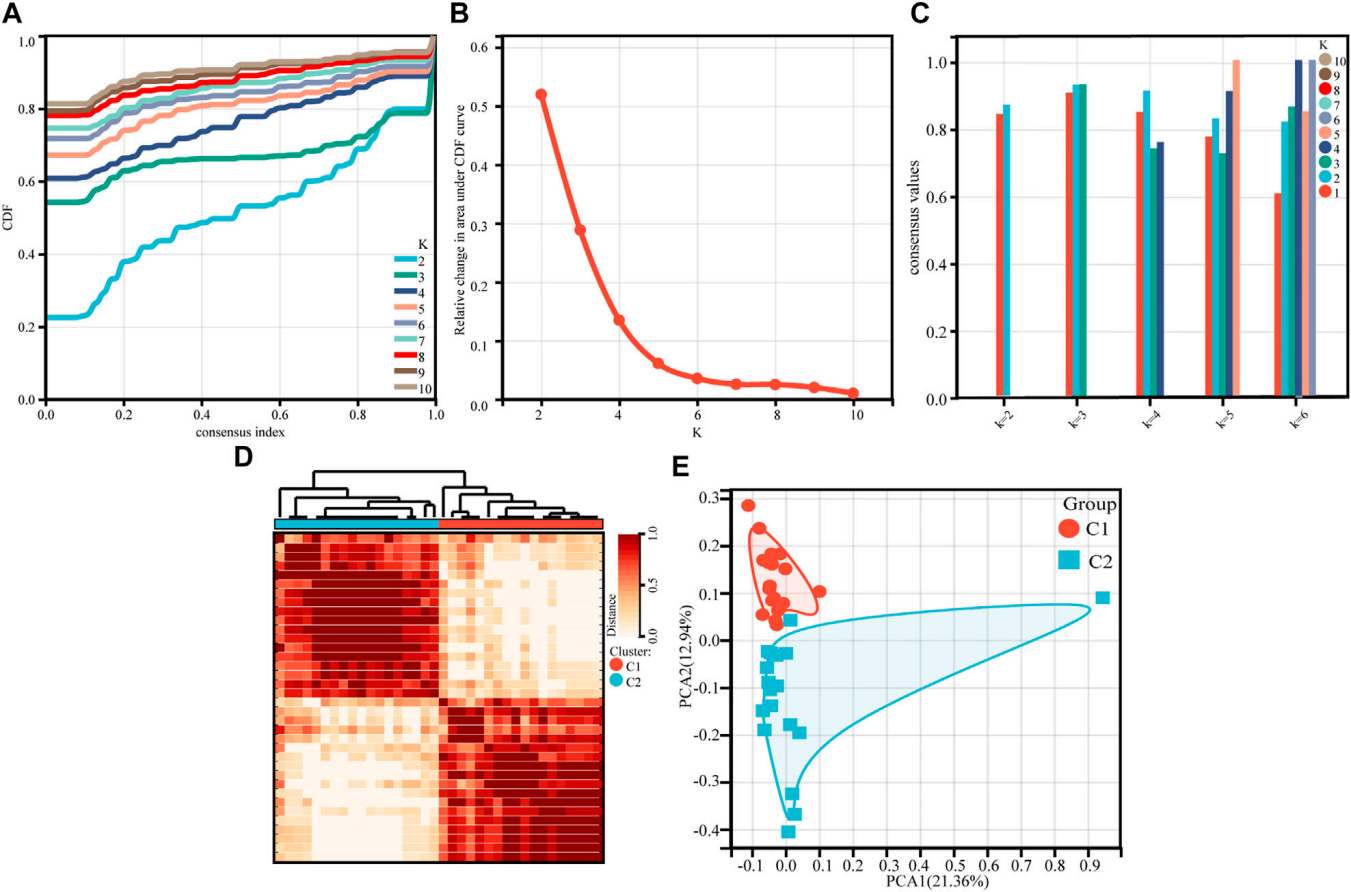
Consensus clustering of gene expression profiles for asthma cases based on the GSE65204. **(A)** Cumulative distribution curves for subtypes with cluster count (k) ranging from 2 to 10. **(B)** Relative change in area under CDF curve for subtypes with cluster count (k) ranging from 2 to 10. **(C)** The bar-plot represents the consensus scores for subtypes with cluster count (k) ranging from 2 to 6. **(D)** Consensus matrix heatmap, which defining two clusters of samples for which consensus values range from 0 (in white, samples never clustered together) to 1 (dark red, samples always clustered together). **(E)** PCA plot of the patients with asthma.

For 36 asthmatic children, an unsupervised cluster analysis was performed. The results showed a high concordance of gene expression patterns in each cluster after 36 children with asthma were divided into two subtypes (18 patients in each subtype) (Figures 1A,B). The consistency analysis of the clustered samples was performed next. The results showed a very high consistency of gene expression patterns within each module when k = 2, and the clustering scores for each subgroup were higher than 0.8 (Figure 1C). The consensus matrix heatmap defined two subtypes of samples for which consensus values ranged from 0 (in white, samples never clustered together) to 1 (dark red, samples always clustered together) (Figure 1D). This indicates that this classification method was more stable than other methods. The PCA analysis revealed that patients in both subtypes were distributed in both directions, confirming the robustness of the clustering results and the differences between the two clusters (Figure 1E). There were no differences in race, age, or gender between the two groups (Table 1).

**TABLE 1:** Demographic characteristics of asthma subjects with different molecular subtypes.

	Group C1 (N = 18)	Group C2 (N = 18)	Total (N = 36)	*p*-value	FDR
Gender				0.5	1
Female	10 (27.78%)	7 (19.44%)	17 (47.22%)		
Male	8 (22.22%)	11 (30.56%)	19 (52.78%)		
Age				0.92	1
10 years	6 (16.67%)	5 (13.89%)	11 (30.56%)		
11 years	6 (16.67%)	6 (16.67%)	12 (33.33%)		
12 years	6 (16.67%)	7 (19.44%)	13 (36.11%)		
Participant race				1	1
Hispanic: No	15 (41.67%)	14 (38.89%)	29 (80.56%)		
Hispanic: Yes	3 (8.33%)	4 (11.11%)	7 (19.44%)		
Participant race				1	1
African American: No	1 (2.78%)	2 (5.56%)	3 (8.33%)		
African American: Yes	17 (47.22%)	16 (44.44%)	33 (91.67%)		
Participant race				0.6	1
White: No	15 (41.67%)	17 (47.22%)	32 (88.89%)		
White: Yes	3 (8.33%)	1 (2.78%)	4 (11.11%)		
Participant race					
Asian: No	18 (50.00%)	18 (50.00%)	36 (100.00%)		
Participant race				1	1
Indian_Alaska: No	17 (47.22%)	17 (47.22%)	34 (94.44%)		
Indian_Alaska: Yes	1 (2.78%)	1 (2.78%)	2 (5.56%)		

### Differential expressed gene screening

**FIGURE 2:**
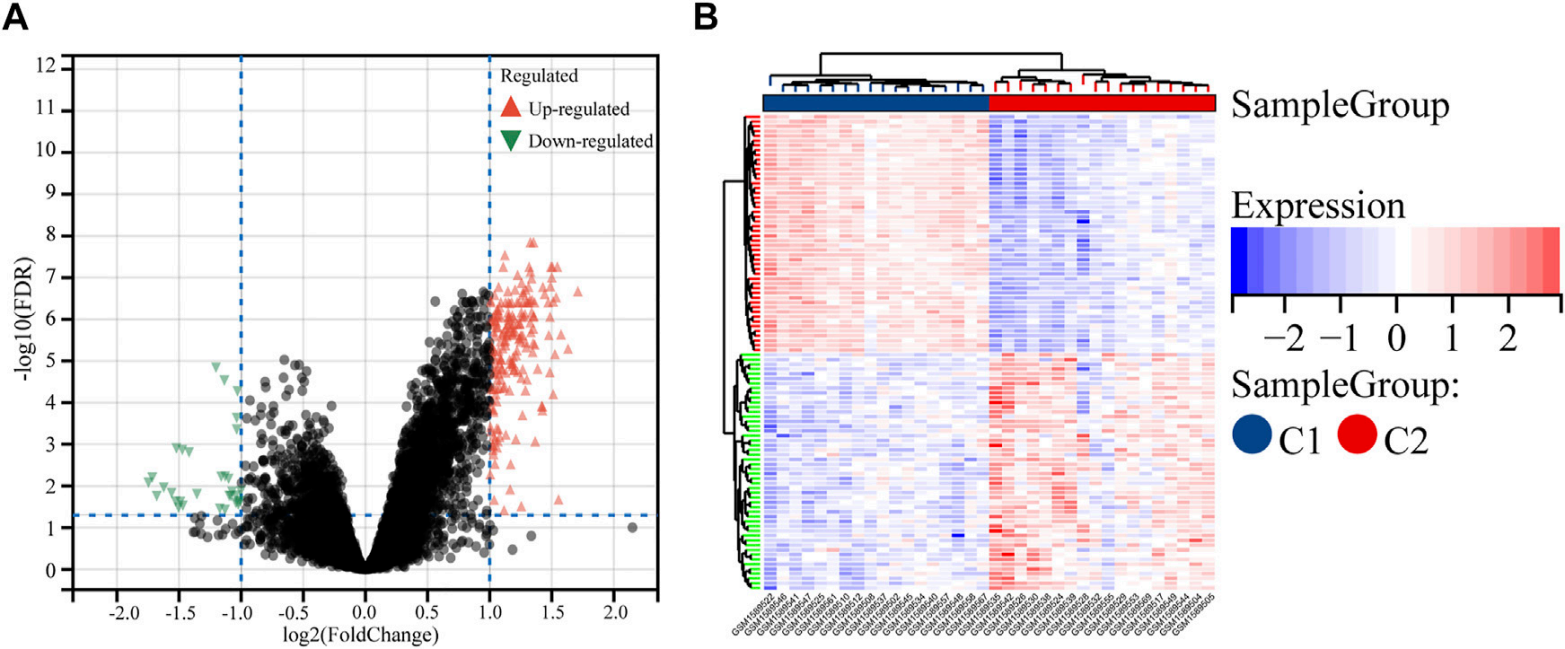
Identification of the DEGs between the two molecular subtypes. **(A)** Volcano plot of the DEGs. **(B)** Heatmap of the DEGs.

Comparing the two subtypes, using |log2FC| > 1 and FDR < 0.05 as the setting conditions, a total of 228 differential genes (196 upregulated and 32 downregulated genes) were identified in C1 compared with C2 (Supplementary Table S2). Their volcano plot and heatmap are shown in the figure (Figures 2A,B).

### Weighted gene co-expression network analysis

#### Weighted co-expression network construction

**FIGURE 3:**
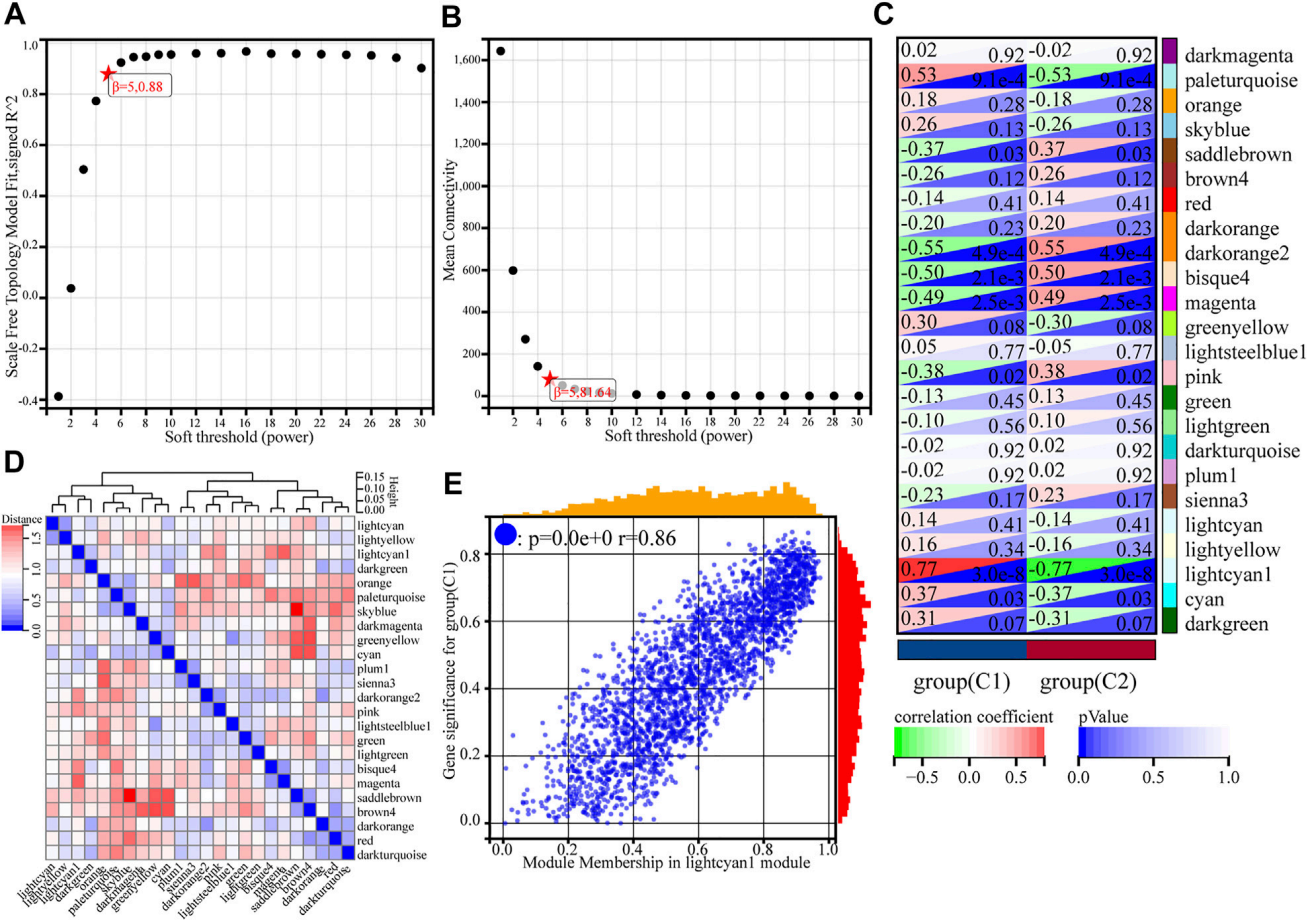
Weighted gene co-expression network analysis of gene expression profiles for asthma cases based on GSE65204. **(A)** Analysis of the scale-free index for various soft-threshold powers (β). **(B)** Analysis of the mean connectivity for various soft-threshold powers. **(C)** Heatmap of the correlation between the module genes and phenotype. **(D)** Heatmap of modular feature vector clustering. **(E)** Correlation between module membership and gene significance, where r denotes the absolute correlation coefficient between GS and MM absolute correlation coefficient.

Using gene expression profiles, the MAD (median absolute deviation) for each gene was first calculated, and then the top 50% of genes with the smallest MAD values were eliminated. Removal of outlier genes and samples was conducted using the goodSamplesGenes method of the WGCNA package in R. The optimal soft threshold (β = 5) was determined when the signed R2 reached 0.88 for the first time (Figures 3A,B). At this point, the average degree of connectivity of the network was relatively high and could contain enough information. The module merge threshold was set to 0.25 to merge modules that were close to each other and similar. In addition, the minimum number of genes for the module was set to 30, the sensitivity was set to 3, and a total of 24 modules were generated (Supplementary Table S3). There were 2413 genes in the lightcyan1 module (Supplementary Table S4), darkorange had 1070 genes, cyan had 384, and red had 214. The grey module was a collection of genes that could not be assigned to any module, so this module was excluded in the next step of the analysis.

#### Critical module identification

To find the co-expression similarity of all modules, the feature genes were calculated based on the correlation between modules. Compared to other modules, the correlation coefficient of the lightcyan1 module and C1 subgroup was 0.77 (*p* = 3.0e-8) (Figures 3C,D). The genes in lightcyan1 module thus may play an important role in the typing of C1 and C2. The picture shows the gene significance (GS) for group C1 and module membership (MM) in the lightcyan1 module correlation coefficient of 0.86 (*p* < 0.0e+0) (Figure 3E). Therefore, the lightcyan1 module is an important module associated with asthma typing. The module has 2413 genes. Setting conditions were as follows: MM is 0.8, GS is 0.2, weight is 0.15, further screening of 551 hub genes in lightcyan1 (Supplementary Table S5).

### Gene ontology and kyoto encyclopedia of genes and genomes pathway analysis

#### GO and KEGG analysis of DEGs

**FIGURE 4:**
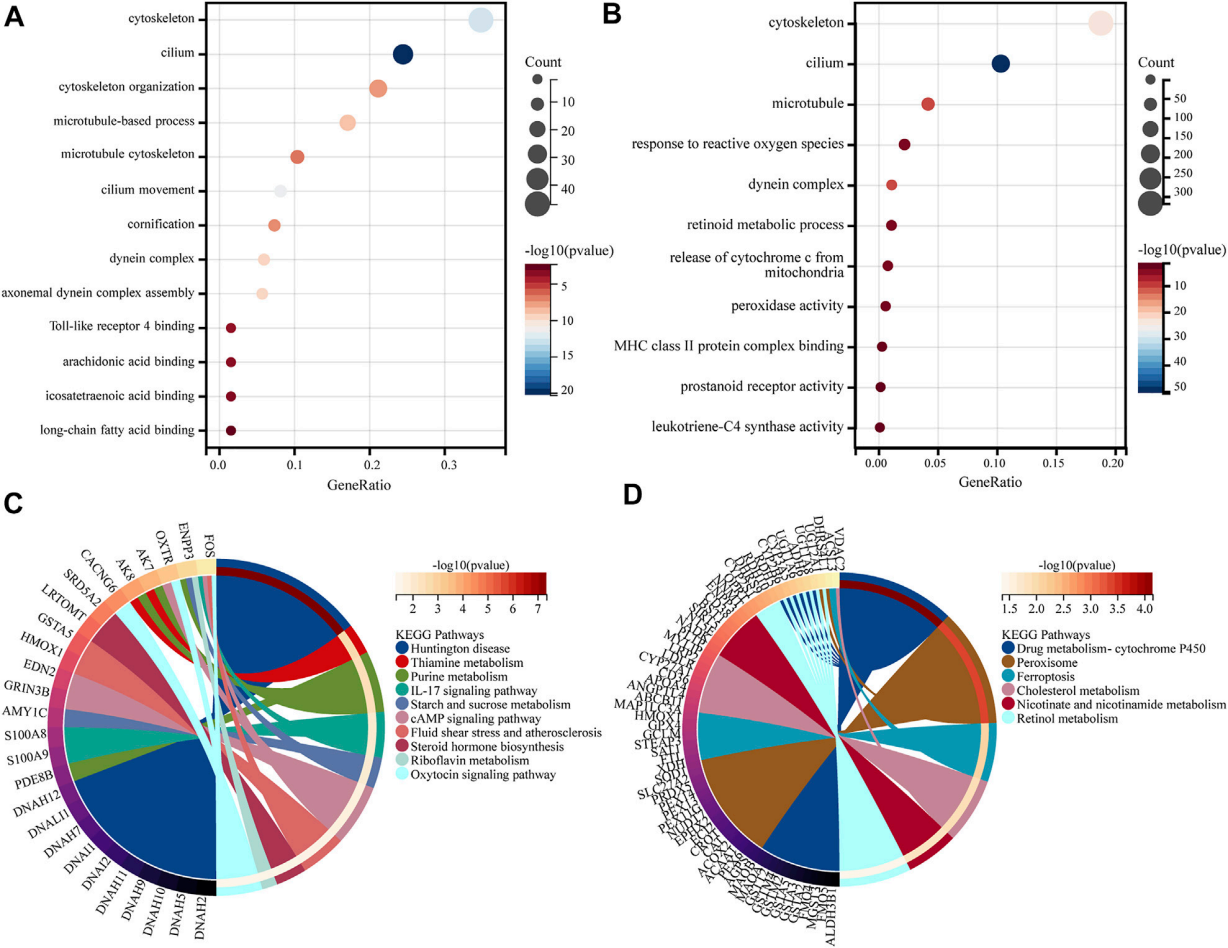
GO and KEGG analysis. **(A)** GO enrichment analysis of genes in the DEGs. **(B)** GO enrichment analysis of genes in the lightcyan1 module. **(C)** KEGG enrichment analysis of genes in the DEGs. **(D)** KEGG enrichment analysis of genes in the lightcyan1 module.

GO and KEGG analysis of DEGs was done using the clusterProfiler package in R. GO functional enrichment analysis and KEGG pathway analysis were both set at *p*-value < 0.05 as a qualifying condition. A total of 431 GO entries were obtained from GO enrichment analysis. The results show that DEGs were primarily related to the cilium (GO: 0005929), cytoskeleton (GO: 0005856), microtubule (GO: 0005874), dynein complex (GO: 0030286), and so on. BP acted mainly through cilium movement (GO:0003341), axonemal dynein complex assembly (GO:0070286), cytoskeleton organization (GO:0007010), microtubule-based processes (GO:0007017), cornification (GO:0070268), etc. The MF aspect mainly involves Toll-like receptor 4 binding (GO: 0035662), arachidonic acid binding (GO: 0050544), long-chain fatty acid binding (GO: 0036041), and icosatetraenoic acid binding (GO: 0050543) functions. The results are shown in Figure 4A. The results of KEGG pathway annotation analysis showed that DEGs are mainly involved in 10 relevant information pathways, including the IL-17 signaling pathway, steroid hormone biosynthesis, cAMP signaling pathway, oxytocin signaling pathway, riboflavin metabolism, thiamine metabolism, purine metabolism, starch and sucrose metabolism, fluid shear stress and atherosclerosis, and Huntington’s disease (Figure 4C).

#### GO and KEGG analysis of lightcyan1 module genetics

The GO and KEGG analysis of 2413 genes was performed in the lightcyan1 Module Genetics using the clusterProfiler package in R. GO functional enrichment analysis and KEGG pathway analysis were both set at *p*-value < 0.05 as a qualifying condition. A total of 1149 GO entries were obtained from GO enrichment analysis. The CC aspect was mainly related to cilium (GO:0005929), cytoskeleton (GO:0005856), microtubule (GO:0005874), dynein complex (GO:0030286), and so on. BP acted mainly through biological processes such as the release of cytochrome c from mitochondria (GO:0090199), response to reactive oxygen species (GO:0000302), and retinoid metabolic process (GO:0001523). The MF aspect mainly involves leukotriene-C4 synthase activity (GO:0004464), prostanoid receptor activity (GO:0004954), peroxidase activity (GO:0004601), MHC class II protein complex binding (GO:0023026), and so on (Figure 4B). The results of KEGG pathway annotation analysis showed that the lightcyan1 module genetics is mainly involved in 32 related information pathways, including drug metabolism-cytochrome P450 (hsa00982), peroxisome (hsa04146), ferroptosis (hsa04216), retinol metabolism (hsa00830), nicotine and nicotinamide metabolism (hsa00760), cholesterol metabolism (hsa04979), and so on (Figure 4D).

### Construction for PPI and screening hub genes

**FIGURE 5:**
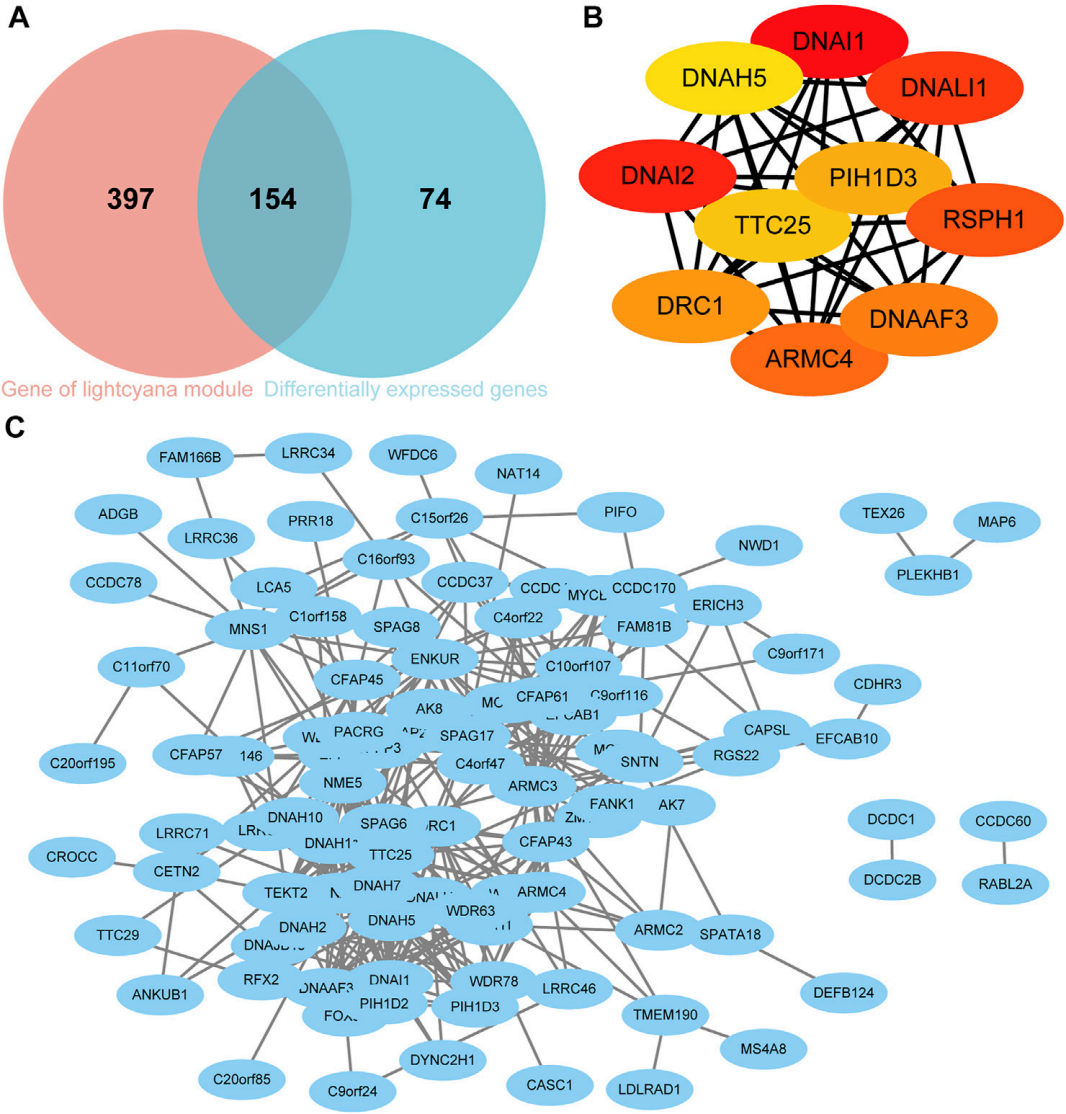
Screening of Hub genes. **(A)** Venn diagram of candidate genes. **(B)** Hub genes. **(C)** Protein-protein interaction (PPI) network based on candidate genes.

The DEGs and hub genes in lightcyan1 were mapped to each other, and 154 candidate genes were obtained (Figure 5A). Subsequently, 154 candidate genes were imported into the STRING database, and the resulting data were imported into Cytoscape to construct the PPI network. A total of 102 nodes and 317 edges were obtained (Figure 5C). Using the MCC algorithm in Cytoscape software’s CytoHubba plugin to screen out the top 10 nodes, namely hub genes, which are DRC1, TTC25, DNALI1, DNAI1, DNAI2, PIH1D3, ARMC4, RSPH1, DNAAF3, and DNAH5 (Figure 5B). All 10 hub genes were upregulated in the C1 group.

### Receiver operating characteristic analysis

**FIGURE 6:**
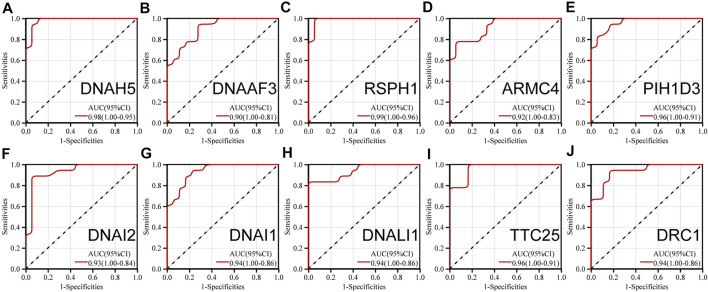
ROC analysis of **(A)** (DNAH5) **(B)** (DNAAF3) **(C)** (RSPH1) **(D)** (ARMC4) **(E)** (PIH1D3) **(F)** (DNAI2) **(G)** (DNAI1) **(H)** (DNALI1) **(I)** (TTC25) **(J)** (DRC1).

The ROC analyses of 10 hub genes were performed separately. The AUC (95% CI) were 0.95 for DRC1, 0.96 for TTC25, 0.94 for DNALI1, 0.93 for DNAI2, 0.96 for PIH1D3, 0.92 for ARMC4, 0.99 for DNAAF3, 0.90 for DNAAF3, and 0.98 for DNAH5 (Figure 6). This indicates that the 10 hub genes can group children with asthma at molecular level.

### Immune cell infiltration analysis

**FIGURE 7:**
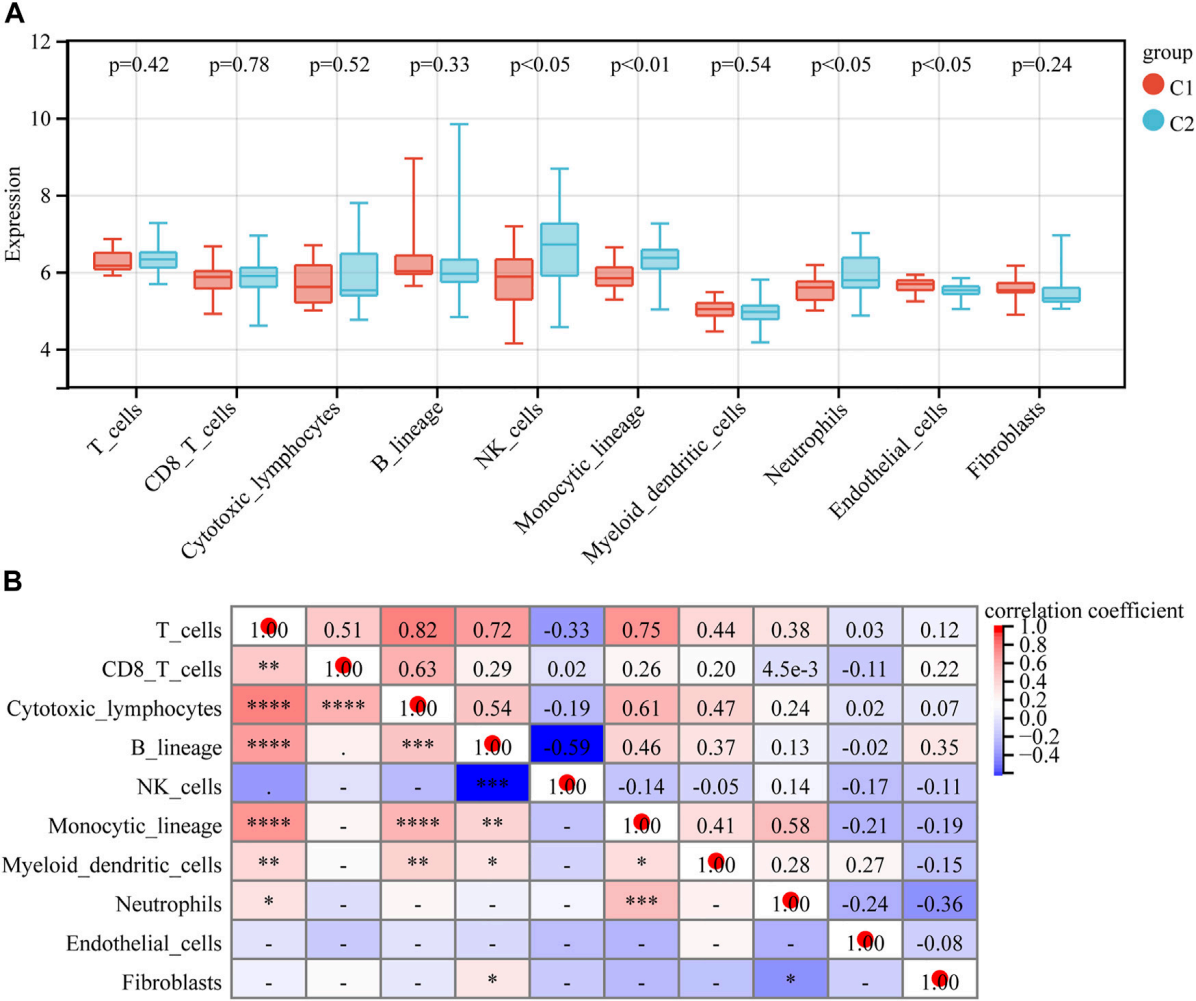
Immune cell infiltration analysis. **(A)** Immune cell and stromal cell infiltration abundance in different subtypes. **(B)** Relevance heatmap of immune cells and stromal cells.

**FIGURE 8:**
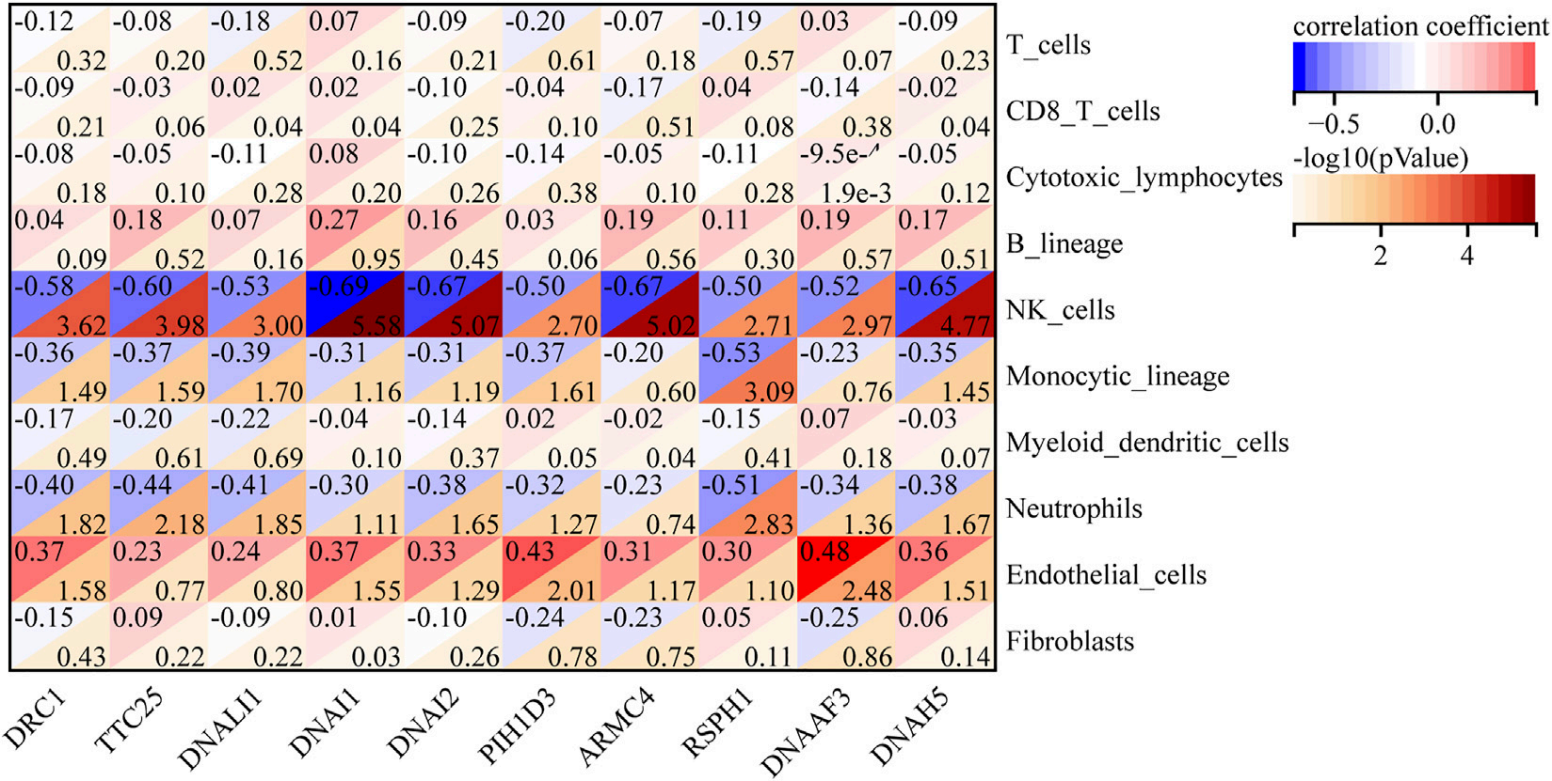
Relevance heatmap of hub genes to immune cells and stromal cells.

The abundance of eight immune cell and two stromal cell populations were assessed using the MCPcounter method in R (Becht et al., 2016b). The abundance of neutrophils, monocytic lineage cells (cells originating from monocytes), and NK cells was significantly higher in C2 than in C1 (*p* < 0.05). However, the abundance of endothelial cells in C1 was significantly higher than in C2 (*p* < 0.05) (Figure 7A). In addition, we found that cytotoxic lymphocytes showed the strongest positive correlation with T cells, with a correlation coefficient of 0.82. NK cells had the strongest negative correlation with B lymphocytes, with a correlation coefficient of −0.59 (Figure 7B). We further analyzed the relationship between 10 hub genes and the regulation of expression in eight immune cells and two stromal cells, finding that 10 hub genes were negatively regulated on neutrophils, monocytic lineage, and NK cells; the 10 hub genes were positively regulated in the endothelial cells (Figure 8).

## Discussion

About 339 million people worldwide are currently affected by asthma, and that number is expected to reach 400 million by 2025 (El-Husseini et al., 2020). Despite its high prevalence, asthma’s pathogenesis remains unclear. Although it is recognized as a heterogeneous disease, there is still no reasonable explanation for it or appropriate classification criteria. Although there are several treatments available worldwide for asthma (Reddel et al., 2022), many of them either have systemic side effects or are ineffective for people with severe asthma. In this study, we analyzed the transcriptional matrix of nasal epithelium. We used unsupervised consensus clustering analysis to classify children with asthma into two subtypes, demonstrating that asthma is a heterogeneous disease. We screened DEGs between C1 and C2 and used WGCNA to obtain critical modules. Our analysis showed differences in gene expression, biological function and immune microenvironment between the two subtypes. Finally, 10 hub genes were screened by constructing PPI. We used ROC analysis to demonstrate the high reliability of these 10 hub genes to group asthma. This paper provides a preliminary exploration of the molecular typing of childhood asthma.

In this study, unsupervised consensus clustering analysis was performed on the GSE65204 dataset in the GEO database. The results of the analysis showed that 36 children with asthma included in the study were divided into two subtypes, C1 and C2, which indicates that a heterogenetic nature for asthma. Furthermore, the specific biological functions and immune status of the two groups of children were analyzed.

The analysis of differences between the two subtypes of children yielded 228 DEGs, including 196 up-regulated genes and 32 down-regulated genes in C1 relative to C2. The GO enrichment analysis of DEGs is mainly related to Toll-like receptor 4 binding, arachidonic acid binding, long-chain fatty acid binding, and icosatetraenoic acid binding. A large number of studies have demonstrated that the above pathways play a role in immune regulation and the release of inflammatory mediators (Ramstedt et al., 1984; Kumar et al., 2016; Leitner et al., 2019; Zhang et al., 2020). Therefore, we speculate that the two subtypes are involved in different immunomodulatory pathways.

KEGG pathway annotation analysis showed that DEGs were mainly involved in IL-17 signaling pathway, steroid hormone biosynthesis, cAMP signaling pathway, oxytocin signaling pathway, and so on. Among them, IL-17 is associated with autoimmunity (Amatya et al., 2017). Thus, we hypothesize that differences in the IL-17 signaling pathway may be critical for asthma typing in children. The phenotype of asthma is regulated by steroid hormones, but the mechanism of its occurrence is not yet explained (Payne and Freishtat, 2012). It has been suggested that cAMP modulates inflammatory response and thus influences the treatment of asthma (Bergantin, 2021). In this study, it was calculated that differences in the cAMP signaling pathway were associated with the differentiation of asthma subtypes, but further experiments are needed to verify whether this is consistent with the above-documented pathways. A study in rats concluded that oxytocin neurons in the paraventricular hypothalamic nucleus increased over time during an asthma attack (Chen et al., 2020). However, there was no further analysis of whether the oxytocin signaling pathway affects the asthma subtype.

GO analysis of the lightcyan1 module gene revealed that they act mainly through biological processes such as the release of cytochrome c from mitochondria, response to reactive oxygen species, and retinoid metabolic processes. Cytochrome c plays an important role in respiration, reactive oxygen species, and apoptosis (Kalpage et al., 2019), so we speculate that it may be related to the pathogenesis of asthma. A number of studies have demonstrated that the occurrence of asthma is associated with reactive oxygen species (Zuo et al., 2013). The present study found that critical model genes are clustered in the response pathway to reactive oxygen species, which provides a new idea for studying the pathogenesis of asthma. Retinoic acid has been found to be involved in asthma relief (Golebski et al., 2021). This may provide new targets for the treatment of asthma.

The results of KEGG pathway annotation analysis showed that the lightcyan1 module genes are mainly involved in 32 related information pathways, including drug metabolism-cytochrome P450, peroxisome, ferroptosis, retinol metabolism, nicotine, and nicotinamide metabolism, cholesterol metabolism, etc. Lynch and Price suggested that the diversity of cytochrome P450 could influence the response to beta-blockers in different children (Lynch and Price, 2007). This may guide the choice of future treatment options for children with asthma. Peroxisome proliferator-activated receptors are an essential component in the pathogenesis of asthma. It has been suggested that this receptor can exert anti-inflammatory effects by inhibiting NF-kB, so it is considered to have anti-inflammatory potential (Kytikova et al., 2020). However, how to induce high expression of this receptor is an unresolved issue at present. Ferroptosis has been a hot research topic in tumor-like diseases in recent years, and some scholars have found that ferroptosis is closely related to the development of acute lung injury and asthma (Xu et al., 2021). This opens up new ideas for future research on the pathogenesis of asthma. It has been suggested that transcriptional modification and cytokine-cytokine receptor interactions of retinol metabolism can induce the production of interleukin-10 (IL-10) + activators of type 2 innate lymphoid cells (ILC2s). IL-10+ ILC2s maintain and repair the integrity of the epithelial barrier and may control Th cell responses (Golebski et al., 2021). Therefore, retinol metabolism may be closely related to the pathogenesis of asthma. A related study (Comhair et al., 2015) in metabolomics found that plasma levels of nicotinamide were significantly higher in children with asthma than in healthy children. This may be related to the pathophysiological explanation of asthma. Cholesterol has a complex effect on inflammatory response, and a US study (Fessler et al., 2009) found that serum total cholesterol and high-density lipoprotein cholesterol levels were significantly lower in asthmatic patients than in non-asthmatic patients; this may be useful for future studies in asthma.

The PPI network obtained in this research has a total of 102 nodes and 317 edges base on 154 candidate genes. The PPI network was further analyzed to yield a total of 10 asthma-typed hub genes include DRC1, DNALI1, DNAI1, DNAI2, DNAH5, TTC25, PIH1D3, ARMC4, RSPH1, and DNAAF3. All 10 hub genes were associated with cilia movement based on previous studies (Mitchison et al., 2012; Hjeij et al., 2013; Wirschell et al., 2013; Onoufriadis et al., 2014; Wallmeier et al., 2016; Paff et al., 2017; Huang et al., 2021; Zur et al., 2021; Bolkier et al., 2022; Lei et al., 2022). In addition, one study found that asthma patients with the RSPH1 mutation had significantly higher levels of nasal-exhaled nitric oxide than other asthma patients (Knowles et al., 2014). The movement of the mucocilium is blocked by a pathological mucin imbalance and an innate immune-depleted proteome that is secreted by IL-13-remodeling epithelial cells. IL-13 is high in type 2 cytokine high asthma (Jackson et al., 2020). Whether this is consistent with the asthma typing in this study requires further study. In addition, this study revealed an interesting phenomenon: all of the 10 hub genes we identified were associated with ciliary dyskinesia syndrome. However, no relationship between ciliary dyskinesia syndrome and asthma has yet been documented.

In this study, immune cell infiltration analysis was used to find that the abundance of neutrophils, monocytic lineage (cells originating from monocytes), and NK cells was significantly higher in the C2 than in the C1. One study found that some children with severe asthma have an abundance of airway neutrophils, increased release of cytokine and chemokine that promote airway responses, more pro-inflammatory macrophages, and a poor response to inhaled corticosteroid therapy (Grunwell et al., 2019). This is very similar to some of our conclusions regarding neutrophils and cells with a monocytic lineage (cells originating from monocytes). We all know that macrophages originate from monocytes. In our findings, the abundance of endothelial cells in C1 was significantly higher than in C2. Endothelial cells play a key role in the transport of eosinophils (Korde et al., 2020). The relationship between C1 and eosinophils asthma deserves our consideration. In addition, we found a worthy phenomenon that the regulation between immune cells and stromal cells in 10 hub genes was completely consistent with the difference between C1 and C2. Is this evidence that the altered microenvironment is the result of hub genes regulation, or is it a coincidence? Further studies are needed to confirm this.

Undeniably, there are some shortcomings in this study. First, it was carried out on the gene expression profiles of nasal epithelial cells from 36 children with persistent asthma, so the sample size of this study was small compared with independent samples and not experimentally validated. Therefore, a large amount of data is needed to verify the conclusions of this paper. Second, the lack of data on the clinical characteristics, treatments, and other biochemical parameters of the asthmatic children in this study limits further analysis of the differences between the two subtypes.

Taken together, this paper uses bioinformatics algorithms to analyze children with persistent asthma, confirming the heterogeneous nature of asthma and indicating the existence of different pathogenesis of asthma, which is particularly important for establishing the molecular-level typing of asthma and targeting the treatment of different types of asthma, especially for children with severe asthma. It is hoped that through the unremitting efforts of scholars, the pathogenesis and treatment of various subtypes of asthma will be clarified, and asthma patients will be free from asthma.

## Data Availability

The original contributions presented in the study are included in the article/Supplementary Material, and further inquiries can be directed to the corresponding author.

## Supplementary material

SUPPLEMENTAL TABLE S1 Clinical data.

SUPPLEMENTAL TABLE S2 Differential expressed genes 2-fold FDR 0.05.

SUPPLEMENTAL TABLE S3 WGCNA module.

SUPPLEMENTAL TABLE S4 Lightcyan1 module gene.

SUPPLEMENTAL TABLE S5 Critical module Identification of lightcyan1.

SUPPLEMENTAL DATA SHEET S1 Expression matrix.
